# Uncovering the pivotal role of MYO6 in myocardial infarction: a multimodally validated diagnostic biomarker and immunotherapeutic target

**DOI:** 10.3389/fimmu.2026.1797028

**Published:** 2026-07-09

**Authors:** Jiawei Wang, Zhiguo Lei, Daiqian Chen, Zhifeng Chen, Liping Qu, Wenjun Zou

**Affiliations:** School of Pharmacy, Chengdu University of Traditional Chinese Medicine, Chengdu, China

**Keywords:** biomarker, immunotherapy, MYO6, myocardial infarction, ScRNA-seq

## Abstract

**Background:**

Myocardial infarction (MI) remains a leading cause of cardiovascular mortality worldwide, underscoring the need for improved diagnostic and therapeutic strategies. Despite recent in clinical management, delayed diagnosis and complications such as adverse myocardial remodeling continue to compromise long-term outcomes. Identifying specific biomarkers and actionable therapeutic targets is therefore crucial for precision medicine in MI.

**Methods:**

We integrated MI-related datasets from the Gene Expression Omnibus (GEO) database and performed differential expression analysis alongside weighted gene co-expression network analysis (WGCNA) to identify key differentially expressed genes (DEGs). Machine learning algorithms were applied to screen diagnostic hub genes and evaluate their diagnostic performance. Two-sample Mendelian randomization (MR) analysis, primarily utilizing the random-effects inverse-variance weighting assessed causal relationships between candidate genes and MI risk. Immune profiling was conducted via immune cell infiltration deconvolution and scRNA-seq analysis. Finally, wet-lab validation was performed using quantitative real-time PCR (RT-qPCR), Western blot, and immunohistochemistry in an *in vivo* animal model.

**Results:**

Our analysis initially identified 15 key diagnostic genes. Through two-sample MR analysis, MYO6 exhibited a significant causal relationship with a reduced risk of MI. Immune infiltration analysis revealed significant enrichment of pro-inflammatory cells (neutrophils and monocytes) and a concomitant depletion of protective immune cells (resting natural killer cells and activated CD4 memory T cells) in MI patients. MYO6 expression correlated positively with protective immune cells and negatively with pro-inflammatory cells. Furthermore, scRNA-seq analysis showed that MYO6 was predominantly enriched in T cells and natural killer cells, potentially regulating their subset differentiation, inhibiting excessive intercellular communication, and promoting collagen-mediated tissue repair, thereby alleviating MI injury and enhancing plaque stability. *In vivo* experiments validated the successful establishment of the MI model, revealing that MYO6 was significantly downregulated at both the mRNA and protein levels in infarcted myocardium.

**Conclusion:**

Through multidimensional analysis of clinical transcriptomic data and experimental validation in animal models, we have identified MYO6 as a potential novel diagnostic biomarker for MI, while highlighting the role of immune homeostasis in disease progression and MYO6 regulation. These findings provide crucial insights for the molecular diagnosis and targeted therapy of MI.

## 1 Introduction

Despite significant advances in early reperfusion, pharmacological interventions and standardised care that have markedly improved the prognosis of myocardial infarction (MI), it remains a major cause of death globally (1, 2). Research confirms that premature atherosclerosis accompanied by plaque rupture or erosion is the most common aetiology, accounting for approximately 90% of adult MI cases (3). The development of MI is a complex, multifactorial process involving multiple pathways. Beyond primary core coronary thrombosis, it encompasses intricate pathophysiological cascades, including inflammatory activation, elevated oxidative stress, cardiomyocyte apoptosis and extracellular matrix remodelling (4, 5). Notably, the immune system plays a central regulatory role in MI pathogenesis. Its dysregulation serves as a pivotal bridge linking atherosclerotic plaque progression and rupture to the subsequent amplified myocardial injury (6, 7). The recruitment and activation of innate immune cells (e.g., macrophages) exacerbate plaque instability and promote thrombosis by releasing pro-inflammatory cytokines and matrix metalloproteinases (8, 9). Concurrently, maladaptive adaptive immune responses (e.g., T cell subset dysregulation) further modulate the inflammatory microenvironment, thereby influencing myocardial repair and fibrotic processes (10).

Although current therapies improve disease control, the complexity and heterogeneity of MI pathogenesis contribute to suboptimal treatment responses and a persistent risk of recurrent adverse events in many patients (11, 12). While coronary angiography remains the diagnostic gold standard, its invasive nature, associated costs and potential complications limit its utility for rapid early screening and dynamic monitoring (13). Cardiac troponin (cTnI/T), despite its high sensitivity and convenience, is primarily valuable for early detection but suffers from limited specificity, as its levels can also be elevated in non-MI conditions such as pulmonary embolism and renal failure (14). Therefore, identifying novel MI biomarkers with high sensitivity and specificity, coupled with the development of precision molecular diagnostics and targeted strategies, is crucial for improving patient stratification and enhancing therapeutic outcomes.

The advancement of multi-omics technologies and bioinformatics tools has established multidimensional integrated analysis as a powerful paradigm for discovering disease-associated molecules from complex datasets. In this study, we integrated heterogeneous information from multi-source datasets through standardised workflows, inter-group differential analysis, functional enrichment annotation and WGCNA. This enabled the preliminary screening of candidate molecules and the systematic analysis of functional pathways. Machine learning algorithms were subsequently employed to construct diagnostic models, thereby refining the gene selection and rigorously evaluating the diagnostic efficacy of these candidate genes. To assess potential causal relationships, Mendelian randomisation (MR) analysis was conducted using expression quantitative trait loci (eQTLs). Furthermore, immune cell infiltration deconvolution and single-cell RNA sequencing (scRNA-seq) analysis were utilized to characterize the immunological features of MI. Finally, experimental validation was conducted to confirm the functional relevance of the identified diagnostic genes. This study aims to uncover novel molecular biomarkers and therapeutic targets for MI, providing a new theoretical foundation and potential strategies for the precision diagnosis, risk stratification, and personalised intervention.

## 2 Materials and methods

### 2.1 Data collection and processing

**Figure 1:**
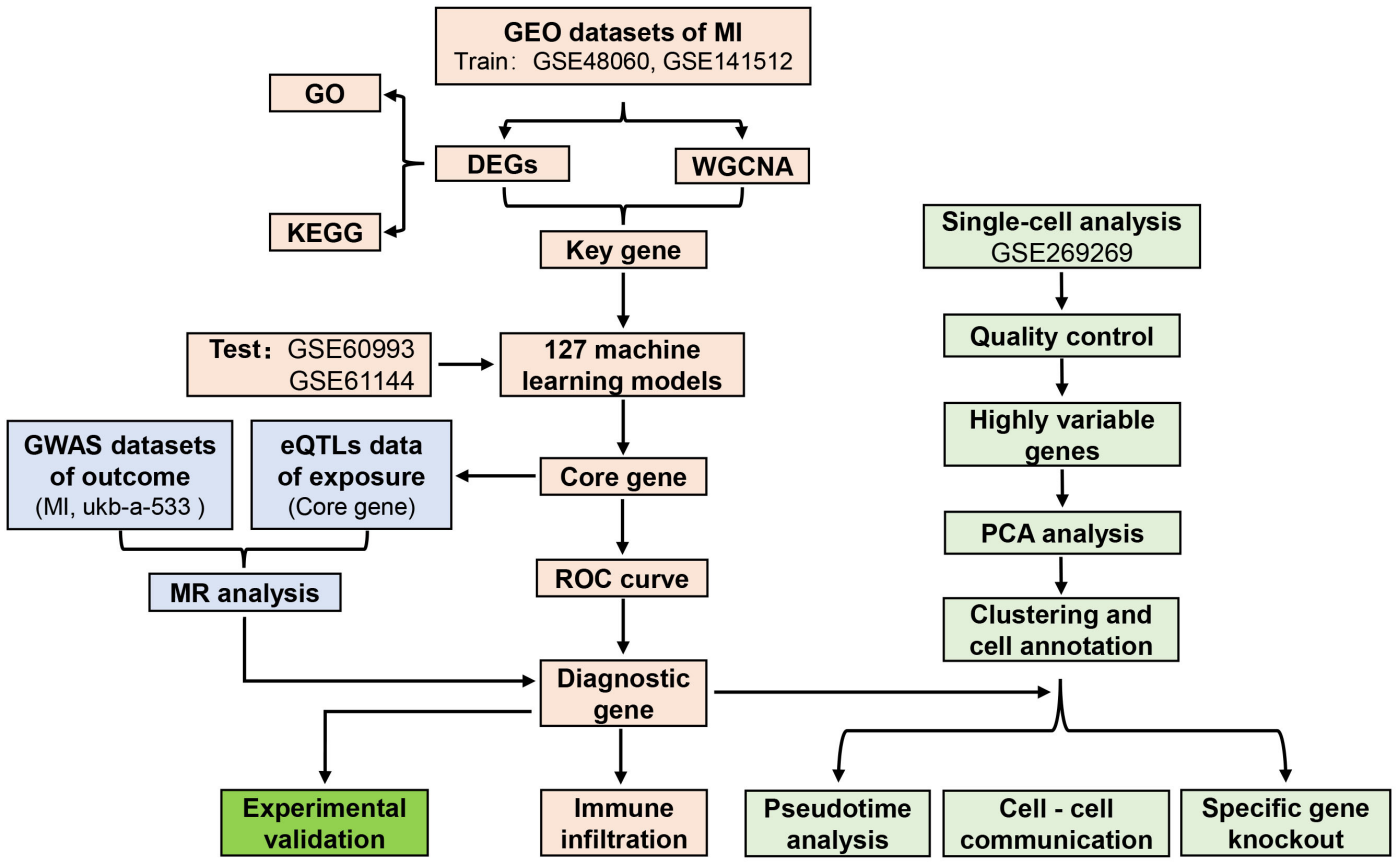
The flowchart of the study.

We first systematically searched the GEO database (http://www.ncbi.nlm.nih.gov/geo) for datasets concerning myocardial infarction. We clearly defined the inclusion and exclusion criteria used when screening public datasets. Inclusion Criteria: (1) The organism must be Homo sapiens; (2) The platform must be expression profiling by arrays; (3) The transcriptomic source must be restricted to human peripheral blood; (4) The dataset must explicitly encompass both myocardial infarction (MI) samples and dynamically matched healthy/normal controls (Con). Exclusion Criteria: (1) Datasets utilizing tissue-level biopsies (e.g., myocardium) rather than peripheral blood; (2) Datasets with an extremely small sample size (total N < 10), which severely compromises statistical power during batch integration; (3) Datasets structurally missing either a standard healthy baseline or clear clinical definitions of MI. Four datasets (GSE48060, GSE141512, GSE60993, and GSE61144) were subsequently selected for downstream analysis. Specifically, GSE48060 and GSE141512 constituted the training set for model construction, while GSE60993 and GSE61144 were employed as the external validation set. Regarding the training cohorts, GSE48060 was generated using the GPL570 platform and comprised peripheral blood samples from 31 MI patients and 21 healthy controls (Con), whereas GSE141512 utilized the GPL17586 platform with 6 MI patients and 6 Con. For the validation cohorts, GSE60993 utilised the GPL6884 platform to analyze 17 MI patients and 7 Con, and GSE61144 employed the GPL6106 platform to analyze 7 MI patients and 10 Con. Batch correction was performed on the datasets using the ComBat method from the “sva” package to mitigate inter-batch technical variations. Principal component analysis (PCA) was conducted both before and after correction to visually assess the mitigation of batch effects. A flow diagram illustrating the overall study design is provided in **Figure 1**.

### 2.2 Identification of DEGs and functional enrichment analysis

Differentially expressed genes (DEGs) between the MI and Con groups were analysed using the ‘limma’ package. DEGs were selected based on an adjusted *P*-value < 0.05 and |logFC| ≥ 0.3. The identified DEGs were visualised using volcano plots and heatmaps generated by the “ggplot2” and “pheatmap” packages. To explore the biological significance of the identified DEGs, functional enrichment analysis encompassing Gene Ontology (GO) and Kyoto Encyclopedia of Genes and Genomes (KEGG) pathways was performed using the ‘clusterProfiler’ package in R. Pathways with an *p* < 0.05 were considered significantly enriched.

### 2.3 WGCNA and module gene selection

To explore the relationship between gene expression patterns and phenotypes, we performed WGCNA using the “WGCNA” package (v1.73) in R. Gene co-expression modules were constructed by selecting an optimal soft thresholding power via the “pickSoftThreshold” algorithm. The power was determined to be 18, achieving a scale-free topology fit index (R² = 0.966). Module identification was performed using hierarchical clustering based on the TOM difference metric (1-TOM), with a minimum module size threshold of 20 genes. Each module was assigned a unique colour identifier. Pearson correlation analysis was employed to assess the relationships between co-expression modules and clinical phenotypes, with the module exhibiting the highest correlation coefficient selected for in-depth investigation. The salmon module demonstrated the strongest negative correlation with the MI phenotype; thus, genes within this module were incorporated into subsequent analyses. To identify disease-critical genes, a Venn diagram analysis was performed to determine the intersection of the DEGs with the core genes from the salmon module.

### 2.4 Diagnostic genes identified by machine learning

To systematically identify diagnostic biomarkers associated with MI, we established a machine learning predictive framework by integrating multiple algorithms. The overlapping genes between the DEGs and WGCNA modules served as the initial candidate diagnostic genes. Subsequently, a total of 127 predictive models were constructed using twelve machine learning algorithms: Lasso, SVM, RF, glmBoost, Stepglm, Ridge, Enet, GBM, LDA, XGBoost, plsRglm, and NaiveBayes. Optimal model parameters were calculated via rigorous cross-validation, with the training cohort partitioned into training and internal validation subsets. Model performance was subsequently assessed using the area under the ROC curve (AUC) across two independent validation datasets (GSE60993 and GSE61144). During model training, the contribution of each gene within every model was documented. Genes consistently identified as major contributors in high-performing models were selected as the diagnostic feature genes. Finally, AUC heatmaps were generated using the “ComplexHeatmap” package.

### 2.5 Identification of the causal relationship between signature genes and MI via MR analysis

#### 2.5.1 Data source

The MR analysis leveraged two primary data sources. Blood-based cis-eQTL data were retrieved from the eQTLGen Consortium (https://eqtlgen.org/) and employed as the exposure variable. MI association data were obtained from the publicly accessible GWAS database of the International EUA Consortium (https://opengwas.io/datasets/). This dataset (GWAS ID: ukb-a-533) comprised 337,199 samples (including 3,927 MI cases and 333,272 controls of European ancestry) and 10,894,596 SNPs, serving as the outcome dataset for our MR analysis.

#### 2.5.2 Instrumental variables selection

In the MR analysis, instrumental variables (IVs) derived from genetic variants are employed to obtain unbiased estimates of the causal effect of the exposure variable on the outcome variable. First, we identified single nucleotide polymorphisms (SNPs) significantly correlated with the exposure variable (*p* < 1×10^−5^) as potential instrumental variables for MR analysis. Linkage disequilibrium (LD) was controlled by setting R² = 0.1 and a LD region width of 500 kb to ensure SNP independence. The strength of the selected instrumental variables was assessed using the F-statistic: F = (N - 2)R²/(1 - R²), where N denotes the sample size and R² represents the proportion of exposure variance explained by the corresponding SNP. An F-value exceeding 10 indicated sufficient statistical power to rule out weak instrument bias, warranting the retention of the instrumental variable.

#### 2.5.3 MR analysis

To evaluate the causal relationship between the exposure and outcome, this study employed the R package “TwoSampleMR” for two-sample MR analysis of instrumental variables and outcome variables. Five distinct MR methods were implemented: inverse variance weighting (IVW), weighted median (WM), MR-Egger, simple mode, and weighted mode. Given the statistical superiority of the IVW method and its consistent estimation of causal effects under the absence of pleiotropy, this approach was adopted as the primary estimator.

#### 2.5.4 Sensitivity analysis

A series of sensitivity analyses were conducted to validate the robustness of the MR findings: (1) Cochran’s Q test was applied under both the IVW and MR-Egger frameworks to assess heterogeneity among IVs; (2) MR-Egger regression was employed to evaluate potential directional horizontal pleiotropy; (3) A leave-one-out was performed by sequentially excluding one SNP at a time and recalculating MR estimates for the remaining SNPs to determine the influence of any single variant on the pooled results.

### 2.6 Immune cell infiltration analysis

The CIBERSORT algorithm was employed to evaluate the infiltration levels of 22 immune cell types between the MI and Con groups. The relative abundances of these immune cell populations were compared across groups and visualized using box plots, with intergroup statistical comparisons conducted via the Wilcoxon rank-sum test. Subsequently, Spearman correlation analysis was performed to investigate the associations between the identified key genes and individual immune cell populations.

### 2.7 Single-cell RNA sequencing analysis

To evaluate the single-cell level expression patterns of the key genes during immune responses, we retrieved peripheral blood scRNA-seq data of 10 MI patients from the GSE269269 dataset, which comprised 5 cases of non-plaque rupture (NPR) and 5 cases of plaque rupture (PR). The “Seurat” R package was employed for downstream single-cell analysis. During quality control, stringent thresholding criteria (200 < nFeature_RNA < 2500 and mitochondrial gene percentage < 20%) were applied to exclude low-quality cells. The retained cellular data were subsequently normalised using the LogNormalise method (scale.factor = 10,000), and the top 2000 highly variable genes were selected via the variance stabilisation transformation (vst) method. Following principal component analysis (PCA)-based dimensionality reduction, unsupervised cell clustering was performed, and umap was utilized for unsupervised clustering and unbiased visualisation of cell subpopulations. The “FindAllMarkers” function package was used to identify differentially expressed genes between clusters. Furthermore, major cell types within each cluster were annotated by consulting the CellMarker 2.0 database and reviewing relevant literature.

Subsequently, the specific cell types exhibiting high expression of the diagnostic genes were extracted and subjected to further subclustering and annotation. The “monocle3” R package was employed for pseudotime clustering to infer differentiation trajectories, state transitions, and dynamic expression patterns of the diagnostic genes in these target cells. To elucidate intercellular signaling, the “CellChat” R package was employed to uncover signaling networks mediated by ligand-receptor pairs between distinct cell subpopulations, thereby revealing differences in cellular communication networks across groups. Subsequently, the “scTenifoldKnk” R package was used for virtual knockout of specific genes, constructing gene regulatory networks to infer the potential functions of the perturbation.

### 2.8 Animal experiments

SPF-grade male SD rats, weighing 220–230 g, were procured from Speifu (Beijing) Biotechnology Co., Ltd. Animals were acclimatised for 7 days in the animal facility at Chengdu University of Traditional Chinese Medicine. All animal procedures were conducted in accordance with the guidelines of the Animal Ethics Committee of CDUTCM and obtained official approval (Approval No.2025079).

SD rats were randomly divided into two groups: blank control (Con, n=6) and myocardial infarction (MI, n=6). MI rats received subcutaneous injections of 85 mg/kg isoproterenol (ISO) (Sigma, USA, Cat.No. I5626-25G), while Con rats received saline, administered once daily at 24-hour intervals for two consecutive days. 24 hours after the final administration, cardiac electrical signal conduction was assessed in each group using a multi-channel physiological signal acquisition system to record heart rate (HR), ST segment, and Q wave changes. Subsequently, high-resolution small animal ultrasound imaging captured short-axis cardiac images. Software analysis determined cardiac function indices including ejection fraction (EF), fractional shortening (FS), stroke volume (SV) and cardiac output (CO). Finally, rats were anaesthetised via intraperitoneal injection of 20% urethane (dose: 0.5 ml/100 g). Whole blood was collected via abdominal aorta puncture. Place the blood sample in a refrigerated high-speed centrifuge and centrifuge at 3,500 rpm for 10 minutes to separate the serum for subsequent CK-MB (Mindray, CHN, Cat.No.141623002) and CK (Mindray, CHN, Cat.No.142523013) level determination. Following whole blood collection from the abdominal aorta, rats were euthanised by decapitation. The heart was rapidly excised and cardiac index calculated (cardiac index = heart weight/body weight × 100%). Subsequently, a segment of cardiac tissue was resected via coronary ring dissection and fixed for subsequent haematoxylin and eosin (HE) staining and immunohistochemical analysis. The remaining cardiac tissue was aliquoted and stored frozen at -80 °C for subsequent molecular analyses.

### 2.9 HE staining and immunohistochemistry

Cardiac tissue from each group of rats was fixed in 4% paraformaldehyde solution, dehydrated, trimmed, embedded, sectioned, stained with HE (Biossci, CHN, Cat.No.BP0211), mounted, and examined microscopically for pathological changes.

For immunohistochemistry analysis, paraffin-embedded cardiac sections were dewaxed and rehydrated, followed by heat-induced epitope retrieval in a citrate buffer. Endogenous peroxidase was blocked at room temperature with 3% hydrogen peroxide, followed by a 30-minute blocking period. Diluted primary antibody MYO6 (1:100, Immunoway, CHN, Cat.No.YT5072) was applied and incubated overnight at 4 °C. Following TBST washing, secondary antibody (1:2000, Abcam, USA, Cat.No.ab205718) was added and incubated at room temperature for 45 minutes. After further washing, DAB (Maxim, CHN, Cat.No.DAB4033) staining and haematoxylin counterstaining were performed. Following dehydration, clearing, and mounting, images were observed under a microscope. Semi-quantitative image analysis was performed using ImageJ software.

### 2.10 RT-qPCR and western blotting

Total RNA was extracted from rat heart tissue using a commercial extraction kit (Foregene, CHN, Cat.No. RE-03014), and RNA concentration was determined using a nucleic acid/protein analyzer. Reverse transcription was performed using 2× RT OR-Easy™ Mix (Foregene, CHN, Cat.No. RT-01022) (42 °C for 15 min; 85 °C for 5 s) to synthesize cDNA. The RT-qPCR reaction system consisted of 2× Blue Real PCR Easy™ Mix-SYBR (Foregene, CHN, Cat.No. QP-03011) and specific primers for MYO6 (Forward primer: ATCCGGTATCTGTCCCAGCA; Reverse primer: GGTTACCCACTATCTCCCCG) and GAPDH (Forward primer: GAAGGTCGGTGTGAACGGAT; Reverse primer: CCCATTTGATGTTAGCGGGAT); the thermal cycling conditions were 95 °C for 3 min, followed by 40 cycles of 95 °C for 10 s and 65 °C for 30 s. GAPDH served as the internal control, and data were analyzed using the 2^−^ΔΔCt method.

Rat cardiac tissue samples were homogenized and thoroughly lysed using RIPA lysis buffer (Beyotime, CHN, Cat.No.P0013B) supplemented with protease and phosphatase inhibitors (Beyotime, CHN, Cat.No.P1045). Protein concentration in the centrifuged supernatant was determined using the BCA protein quantification kit (Beyotime, CHN, Cat.No.P0010S). Samples were uniformly mixed with 5× Protein Loading Buffer (Beyotime, CHN, Cat.No.P0015L) at a 1:4 ratio and heated at 100 °C for 5 minutes to denature the proteins. Proteins were separated by a 7.5–12.5% SDS-PAGE gel (Epizyme, CHN, Cat.No.PG222) and transferred onto a PVDF membrane. The PVDF membrane were then blocked with rapid blocking solution (Epizyme, CHN, Cat.No.PS108P) at room temperature for 30 minutes, followed by washing in TBST buffer. The primary antibody was diluted using universal antibody dilution Buffer (Ncmbio, CHN, Cat.No.WB-100D). The washed PVDF membrane was incubated overnight at 4 °C with primary antibody MYO6 (1:1000 dilution). The following day, the PVDF membrane was washed three times with TBST buffer and incubated at room temperature for 1 hour with the secondary antibody, HRP-labelled goat anti-rabbit IgG (1:500,000). Following incubation, the membrane was washed three times with TBST buffer, enhanced chemiluminescent substrate was added, and the membrane was then imaged using an intelligent gel imaging system. Grayscale values were analysed using ImageJ software. To detect the internal control, the PVDF membranes were rinsed with TBST for 5 minutes, and an antibody stripping buffer (Epizyme, CHN, Cat.No.PS107) was added to remove primary and secondary antibodies. The membrane was then re-blocked with rapid blocking solution for 30 minutes, washed, and treated with GAPDH (Huabio, CHN, Cat.No.ET1601-4) as the internal control, following the same primary and secondary antibody incubation procedures described above.

### 2.11 Statistical analysis

Data are expressed as mean ± SD. Statistical analyses and visualization were performed with SPSS (version 27) and GraphPad Prism (version 10.1.2). Comparisons between two groups were performed using the two-tailed Student’s t test for normally distributed data or Mann–Whitney rank sum test for non-normally distributed data. A *p*-value < 0.05 was considered statistically significant.

## 3 Results

### 3.1 Identification of DEGs and functional enrichment analysis

**Figure 2:**
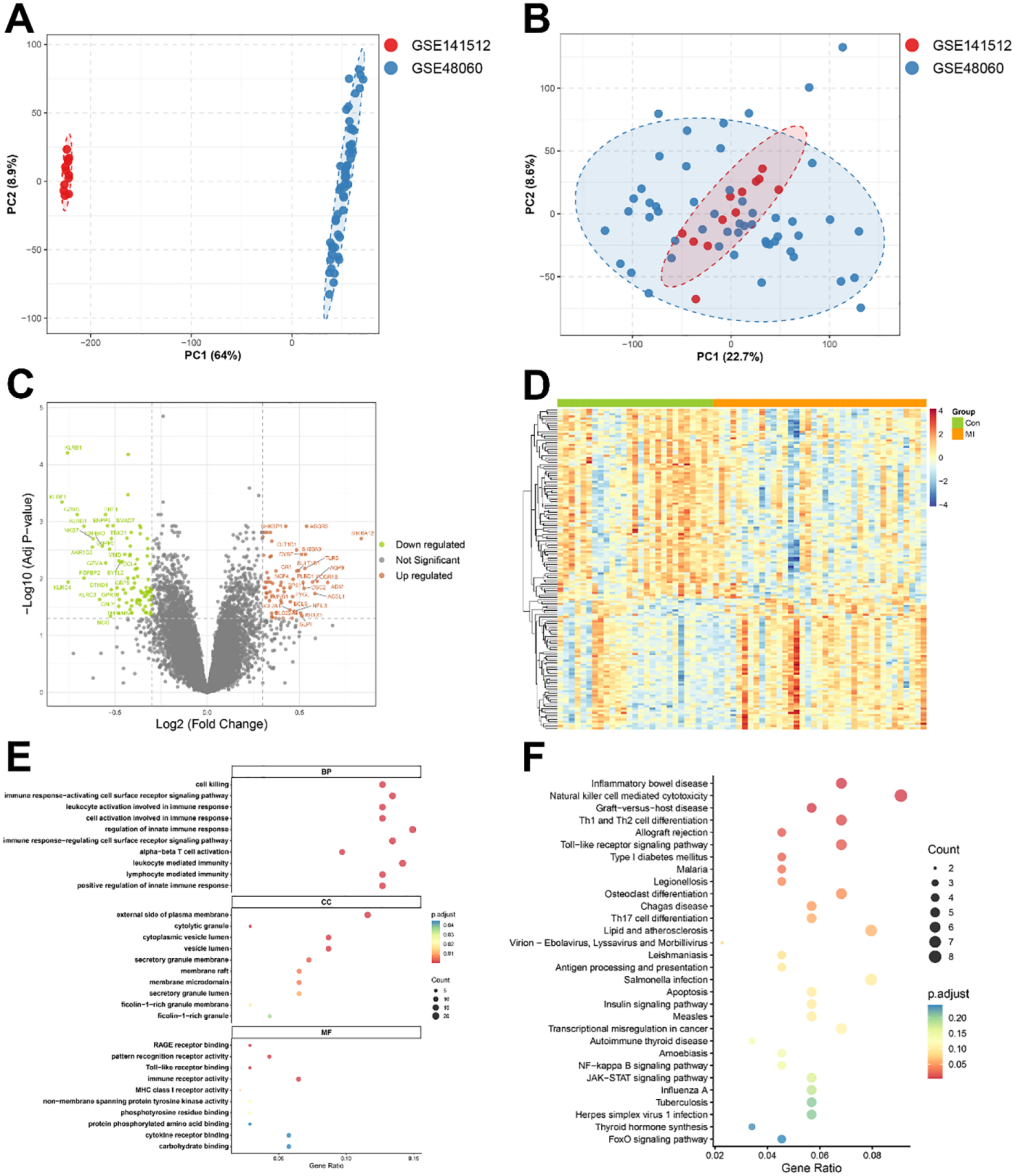
Identification of DEGs and functional enrichment analysis. **(A, B)** Display the principal component analysis plots before and after batch effects were removed; **(C)** Volcano plot shows the DEGs with the greatest fold change between groups; **(D)** Heatmap plot displays the expression patterns of all DEGs between groups. **(E)** Bubble plot displays the top 10 most significant items across the three categories of GO analysis; **(F)** Bubble plot displays the top 30 most significant pathways from the KEGG results.

The training dataset was established by merging the GSE48060 and GSE141512 cohorts. Following normalization and batch effect correction, PCA demonstrated effective sample integration and the elimination of batch effects across the different datasets, thereby validating the multi-platform integration (**Figures 2A, B**). The final merged dataset comprised 64 samples (including 27 Con and 37 MI samples). Differential expression analysis identified 147 DEGs, with 60 upregulated and 87 downregulated in the MI samples (**Figures 2C, D**). Functional enrichment analysis revealed that these DEGs primarily participate in biological processes (BP) such as cell killing, the immune response-activating cell surface receptor signaling pathway, and leukocyte activation involved in the immune response; key molecular functions (MF) included RAGE receptor binding, pattern recognition receptor activity, Toll-like receptor binding, and immune receptor activity (**Figure 2E**). At the pathway level, KEGG analysis revealed significant enrichment in Natural killer cell-mediated cytotoxicity, Th1 and Th2 cell differentiation, and Toll-like receptor signaling pathways (**Figure 2F**). These findings indicate that during myocardial infarction, the identified DEGs primarily participate in disease progression by regulating immune cell-associated expression networks.

### 3.2 Identification of MI-associated gene modules through WGCNA

**Figure 3:**
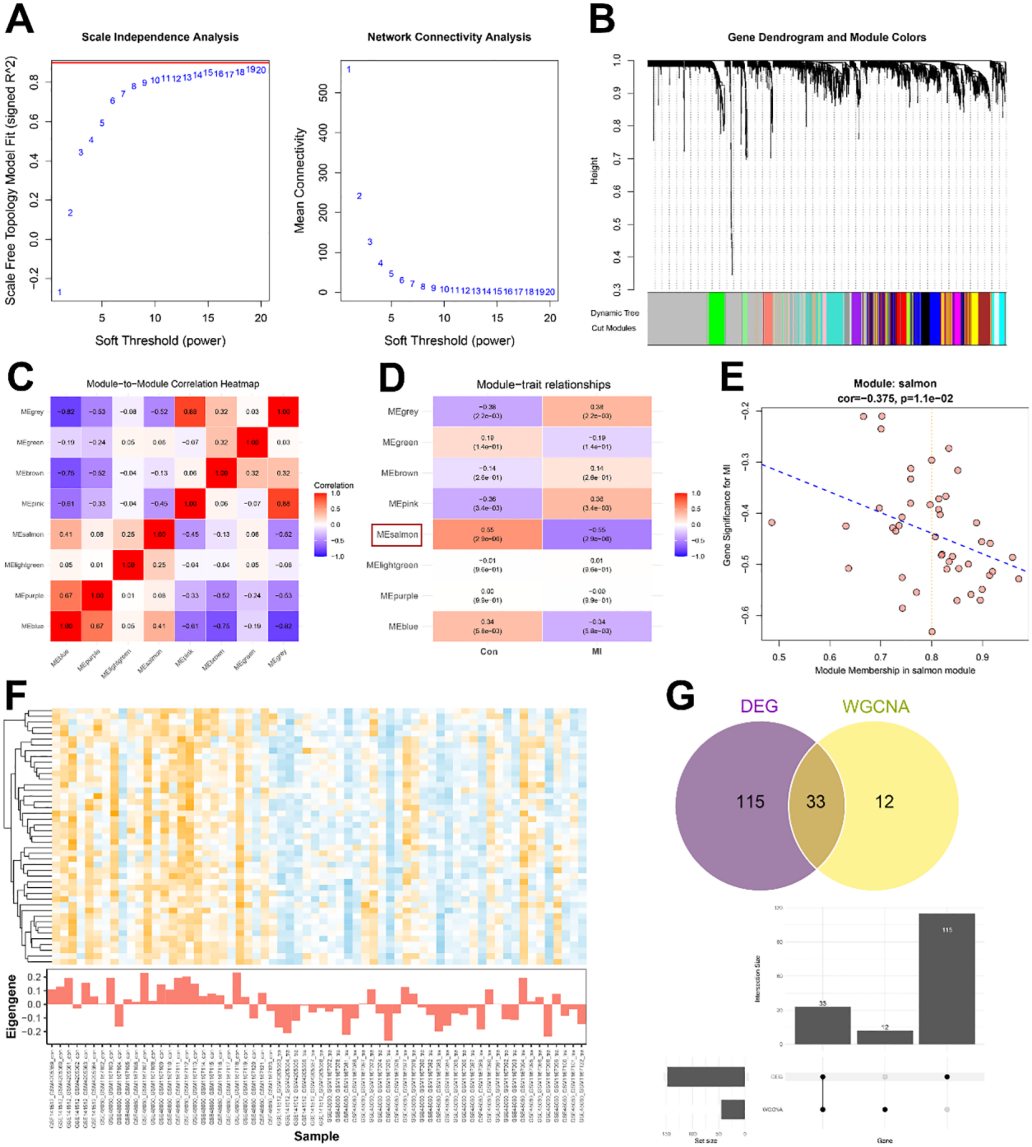
WGCNA analysis of co-expression modules associated with MI and identification of key modules. **(A)**. Soft threshold selection analysis; **(B)**. Construction of gene clustering tree based on gene expression-derived TOM; **(C)**. Inter-module correlation heatmap: displays Pearson correlation coefficients of eigenvalues for each co-expression module, with colour intensity indicating correlation strength; **(D)**. Module-phenotype correlation heatmap displaying the correlation coefficients between each module’s eigenvalue and MI; **(E)**. Scatter plot of gene significance (GS) versus module membership (MM) for salmon modules; **(F)**. Gene expression heatmap and eigenvalue distribution for salmon modules; **(G)**. Venn diagram showing the intersection of DEGs and WGCNA module genes.

The expression patterns of MI-associated genes were further explored via WGCNA. Soft-threshold power selection determined the optimal threshold of 18 to achieve a scale-free topology network (**Figure 3A**). A gene clustering dendrogram was constructed based on the topological overlap matrix derived from gene expression, yielding multiple colour-coded co-expression modules (**Figure 3B**). An inter-module correlation heatmap revealed varying degrees of association among module eigengenes (**Figure 3C**). Furthermore, module–trait correlation analysis further confirmed the salmon module exhibited significant negative correlation with MI phenotype (cor = -0.55, *p* = 2.9e-06) (**Figure 3D**). Notably, among all identified modules, the salmon module displayed the highest absolute correlation coefficient and the most robust statistical significance, indicating its tightly bound regulatory role in MI pathology. This strong negative correlation suggests that the expression of genes within the salmon module is characteristically suppressed during the progression of MI, potentially reflecting the impairment of essential endogenous protective or metabolic pathways. Correlation analysis between gene significance and membership scores within the salmon module revealed a significant negative relationship, indicating that the genes in this module are strongly associated with myocardial infarction (**Figure 3E**). The gene expression heatmap and eigenvalue distribution of the salmon module further illustrated its specific expression pattern in MI samples (**Figure 3F**). A Venn diagram indicated an overlap of 33 genes between this module and the DEGs (**Figure 3G**), demonstrating that these genes constitute both core members of the differential expression profile and key components of the co-expression network. Consequently, they may exert a central regulatory role during MI.

### 3.3 Identification of MI diagnostic gene features, performance evaluation

**Figure 4:**
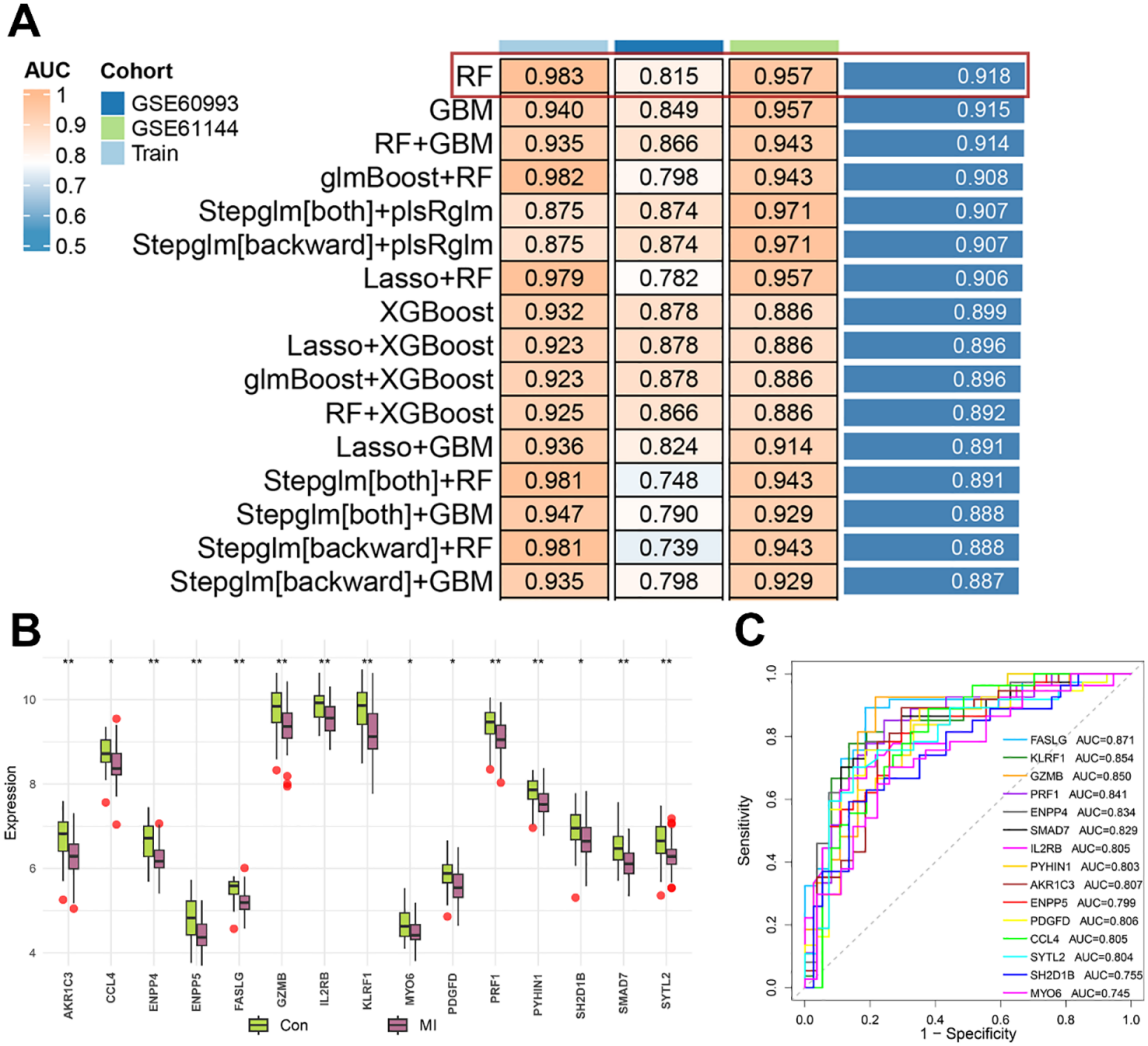
Performance evaluation of MI prediction models. **(A)**. Heatmaps and quantitative results for the top 16 out of 127 predictive models; **(B)**. Box plot of expression distribution for diagnostic genes in the training set; **(C)**. ROC curve for diagnostic genes in the training set.

To construct a predictive model for MI, this study employed 12 machine learning algorithms to build 127 classification models. The results indicated that the Random Forest (RF) model demonstrated the most favorable overall performance (mean AUC value of 0.918) (**Figure 4A**; **Supplementary Figure S1**). This optimal model comprised 15 signature genes: FASLG, KLRF1, GZMB, PRF1, ENPP4, SMAD7, IL2RB, PYHIN1, AKR1C3, ENPP5, PDGFD, CCL4, SYTL2, SH2D1B, and MYO6. The differential expression of these signature genes between the MI and normal samples is illustrated in **Figure 4B**, while their individual diagnostic performance was validated through ROC curve analysis on the training set (**Figure 4C**).

### 3.4 Causal effects of genetically predicted eQTLs on MI and identification of candidate genes

**Figure 5:**
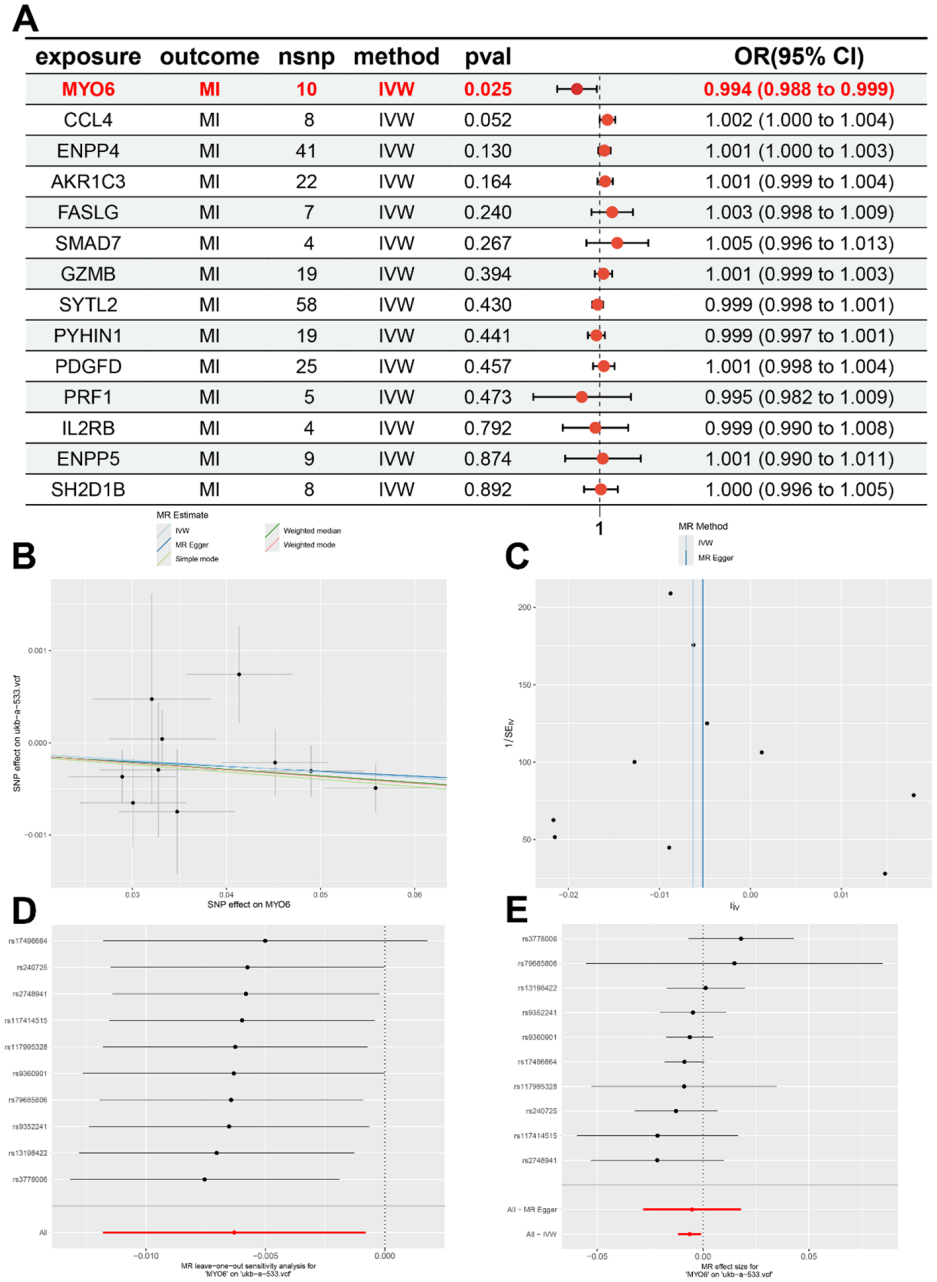
Bimodal MR analysis of diagnostic genes and MI with sensitivity validation. **(A)**. MR results forest plot displaying IVW effect sizes for 14 genes associated with MI; **(B)**. MR-Egger scatterplot assessing horizontal pleiotropy; **(C)**. Funnel plot evaluating instrumental variable heterogeneity; **(D)**. Leave-one-out sensitivity analysis plot; **(E)**. MR-Egger pooled effect forest plot.

A two-sample MR analysis was performed to examine the causal associations between the 15 diagnostic genes and MI. The results indicated that only MYO6 exhibited a significant causal association with MI according to the IVW method (*p* = 0.025, OR = 0.994, 95% CI: 0.988–0.999), suggesting increased MYO6 expression exerts an inhibitory effect against MI (**Figure 5A**). **Supplementary Table 1** summarises the detailed statistical results (including effect estimates, 95% CIs and p-values) obtained using all five MR methods for each gene. For MYO6, the direction of the causal effects estimated by the five MR methods was largely consistent, and the effect sizes were comparable; this confirms that the conclusions drawn from the primary IVW method are highly consistent with those from the other four analytical methods. Sensitivity analyses revealed no evident pleiotropic effects in the MR-Egger regression scatterplot, while funnel plots indicated low heterogeneity among instrumental variables (SNPs) (**Figures 5B, C**). Furthermore, leave-one-out analysis confirmed that excluding individual SNPs did not substantially alter the MR findings, underscoring the robustness of the results (**Figure 5D**). A MR meta-analysis forest plot visualised the association between MYO6 and MI (**Figure 5E**). These MR results demonstrate a robust causal association between MYO6 and MI.

### 3.5 Establishment of MI models and validation of MYO6 expression

**Figure 6:**
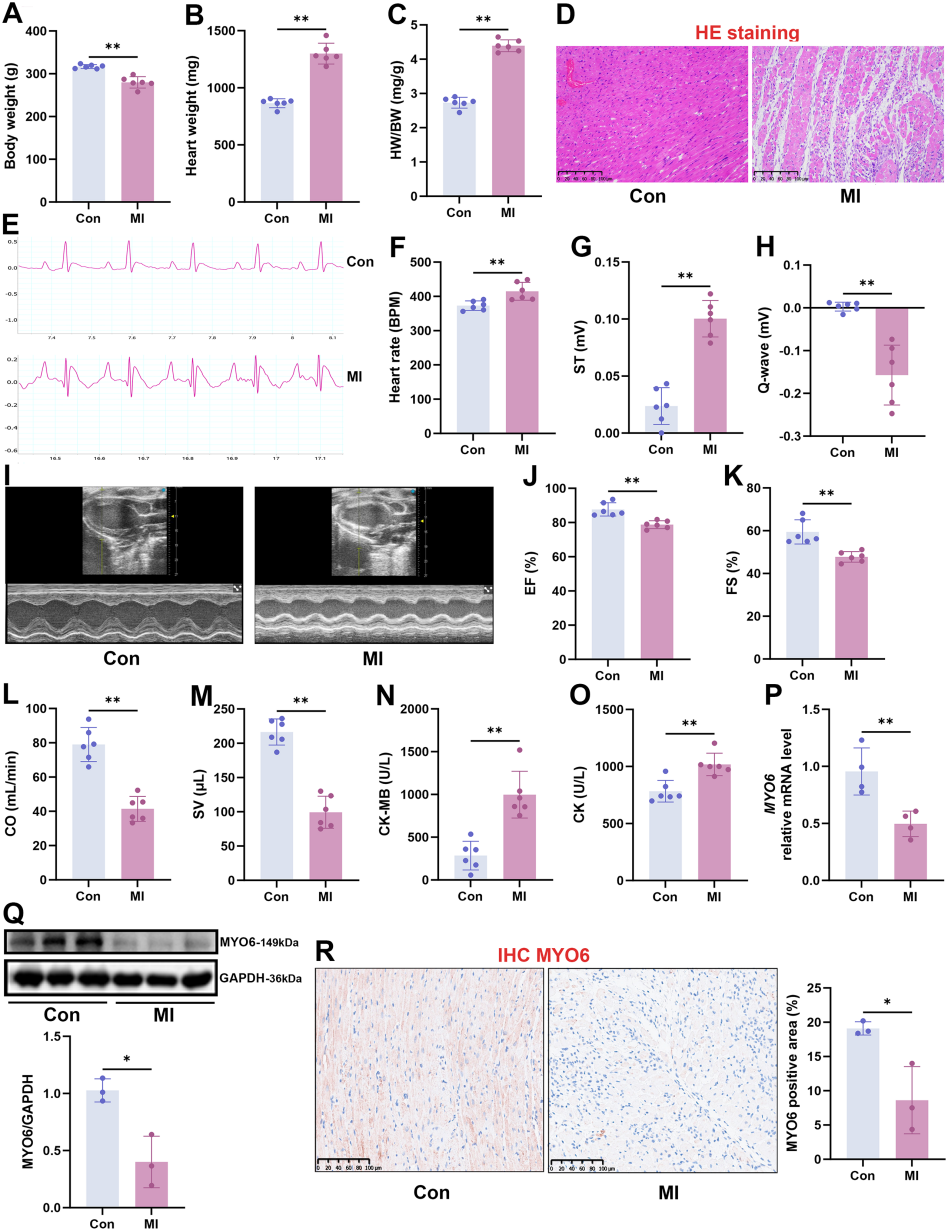
Establishment of myocardial infarction models and validation of MYO6 expression. **(A–C)** Levels of body weight, heart weight and heart weight/body weight (HW/BW); **(D)** HE staining of cardiac tissue; **(E–H)** Representative electrocardiogram images and levels of heart rate, ST-segment elevation, and Q wave amplitude; **(I–M)** Representative echocardiographic images and levels of EF, FS, CO, and SV; **(N, O)** Levels of serum biochemical indicators (CK-MB, CK); **(P)** MYO6 relative mRNA level; **(Q)** Representative WB images and relative protein expression levels of MYO6; **(R)** Representative immunohistochemical images and percentage of MYO6-positive area. *P < 0.05; **P < 0.01.

To elucidate the expression characteristics of MYO6 in MI, we first established an MI animal model. The results demonstrated that the body weight, heart weight, and the heart weight/body weight ratio (HW/BW) in the MI group exhibited significant alterations compared with the Con group (**Figures 6A–C**); HE staining of the cardiac tissue revealed a disorganized myocardial structure in the MI group, which markedly differed from the normal myocardial structure observed in the Con group (**Figure 6D**). Electrocardiographic monitoring demonstrated typical myocardial infarction abnormalities in the MI group, including heart rate, ST segment elevation, and Q wave amplitude (**Figures 6E–H**). Analysis of cardiac function indicators revealed that the EF, FS, CO, and SV were significantly lower in the MI group than in the Con group (**Figures 6I–M**). Serum cardiac enzymes CK-MB and CK were also markedly elevated in the MI group (**Figures 6N, O**), indicating severe myocardial injury. Furthermore, validation of MYO6 expression revealed that its relative mRNA levels, protein expression, and the proportion of immunohistochemically positive areas were significantly downregulated in the MI group compared with the Con group (**Figures 6P–R**). Collectively, these findings confirm the successful establishment of the MI model, and are consistent with our previous multidimensional analysis, confirming that the expression of MYO6 is significantly reduced during MI.

### 3.6 Analysis of MYO6 immune infiltration

**Figure 7:**
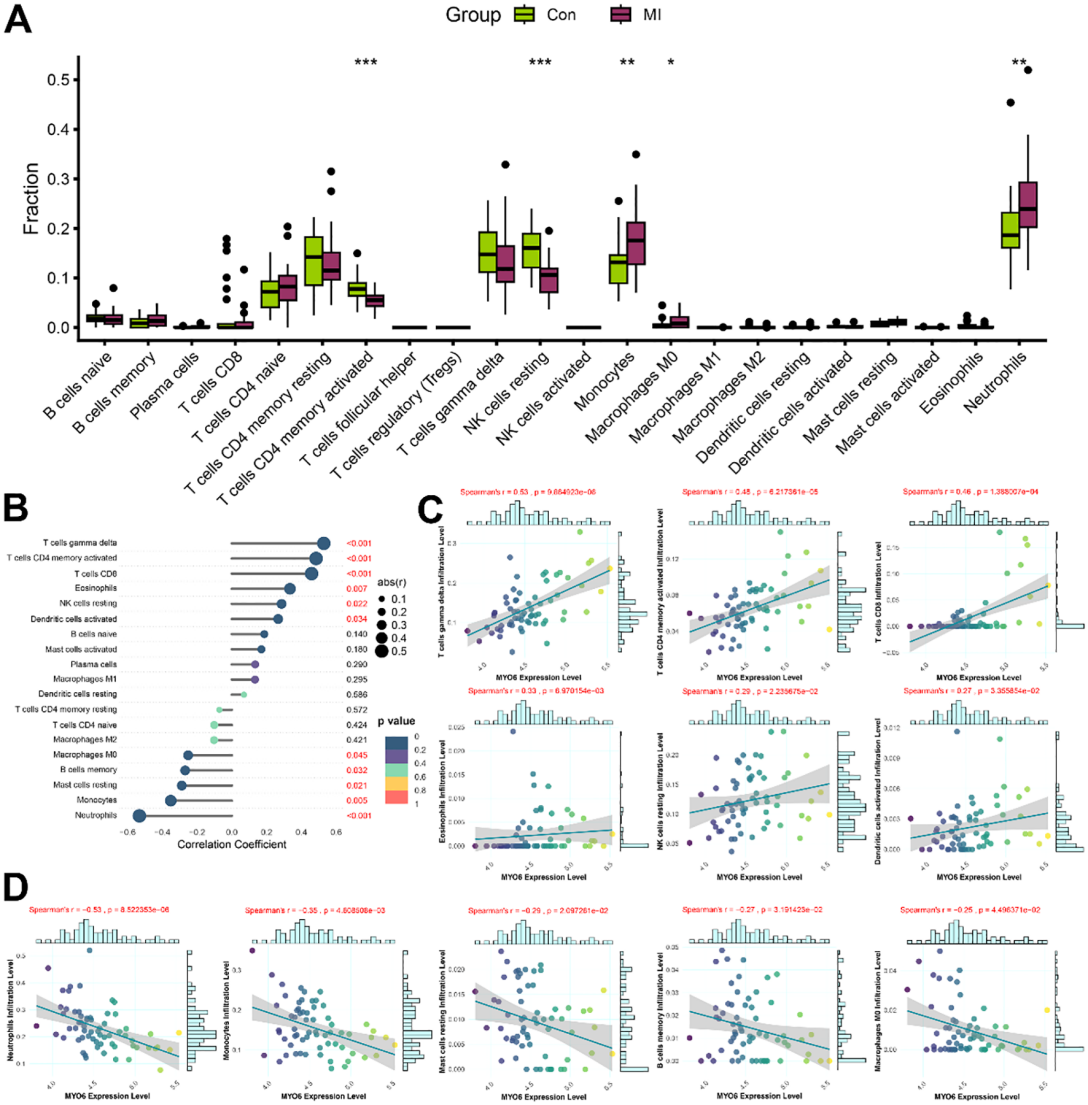
Analysis of immune infiltration. **(A)** Comparison of immune cell infiltration between MI and Con; **(B)** Correlation between MYO6 expression and different immune cell infiltration; **(C, D)** Showed immune cells significantly associated with MYO6 expression. Compared to Con group, *0.01 ≤ *p* ≤ 0.05, **0.001 ≤ *p* ≤ 0.01, ***p < 0.001.

Given the pivotal role of immune responses in MI, we employed the CIBERSORT algorithm to analyze immune infiltration analysis. The findings revealed a marked increase in Neutrophils, Monocytes, and Macrophages M0 cells among MI patients, alongside substantial reductions in resting NK cells and memory activated CD4 T cells (**Figure 7A**). In MI patients, MYO6 expression exhibited significant positive correlations with T cells CD8, T cells CD4 memory activated, T cells gamma delta, NK cells resting, and Dendritic cells activated, whilst showing negative correlations with B cells memory, Monocytes, Macrophages M0, Mast cells resting, and Neutrophils (**Figures 7B–D**).

### 3.7 Single-cell RNA sequencing and gene regulatory network analysis

**Figure 8:**
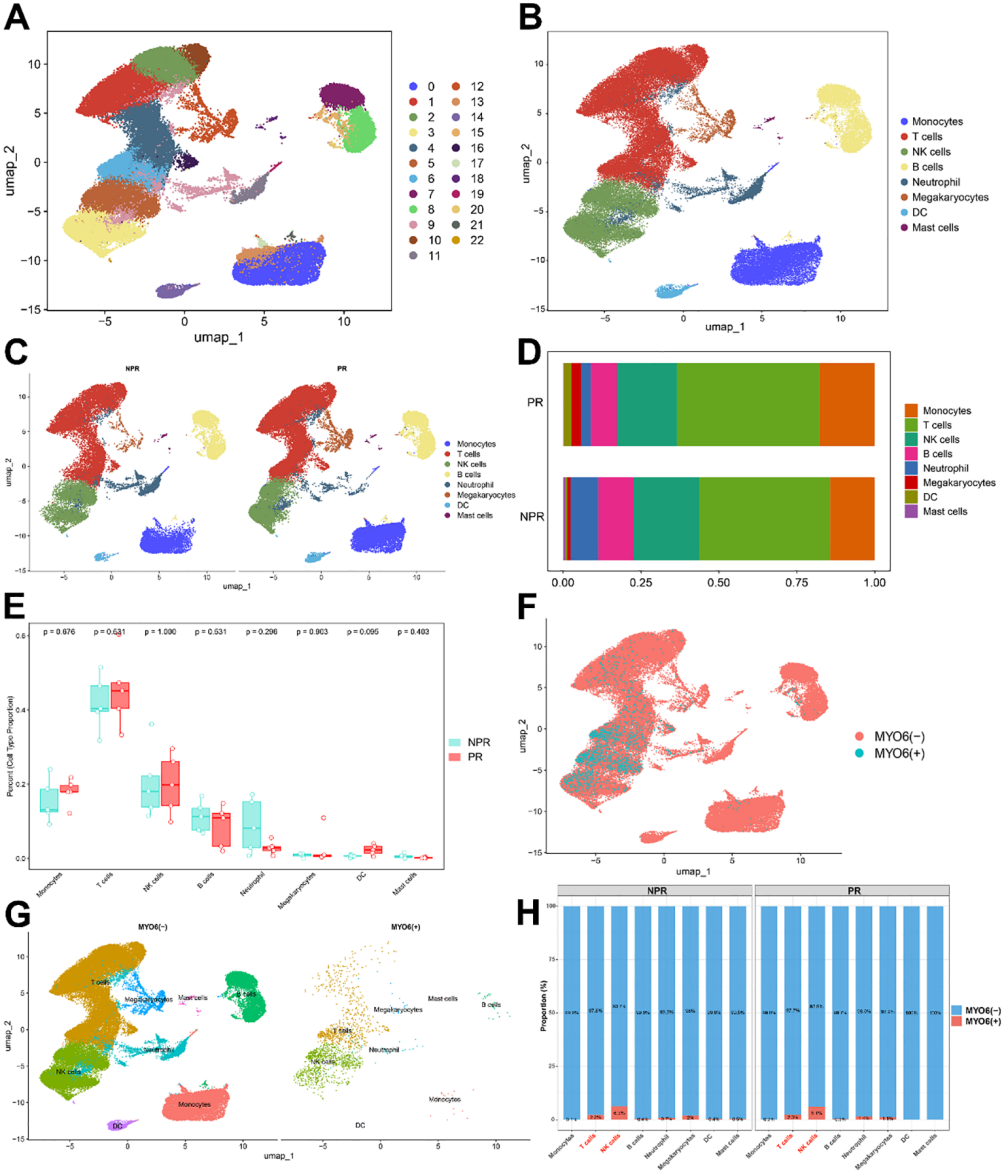
Single-cell transcriptomic analysis of peripheral blood in MI. **(A)** UMAP plot displaying all aggregated single cells; **(B)** UMAP plot showing cell annotation results; **(C)** UMAP plot comparing the distribution of different cell types between the NPR and PR groups; **(D)** Stacked bar chart illustrating the proportion of different cell types in the NPR and PR groups; **(E)** Proportional differences in cell types between NPR and PR groups; **(F)** UMAP plot distinguishing MYO6(-) and MYO6(+) cell distributions; **(G)** UMAP plot comparing cell type distributions within MYO6(-) and MYO6(+) cell populations; **(H)** Bar chart illustrating the proportion of MYO6(-) and MYO6(+) cells within different cell types in the NPR and PR groups.

To thoroughly investigate the immunological response patterns of characteristic genes in MI, alongside their associated cellular functions and regulatory networks, we leveraged single-cell RNA sequencing (scRNA-seq) data derived from the peripheral blood of MI patients. Unsupervised clustering categorized the global cellular landscape into 23 distinct clusters, which were further annotated into eight major lineages: Monocytes, T cells, NK cells, B cells, Neutrophils, Megakaryocytes, DCs, and Mast cells (**Figures 8A, B**). Quantitative compositional analysis revealed that T cells and NK cells constituted the predominant immune cell populations in the peripheral blood of both non-ruptured plaque (NPR) and ruptured plaque (PR) patients (**Figures 8C, D**), showing stable relative proportions across groups (**Figure 8E**). Crucially, when mapping MYO6 expression onto the UMAP space, we observed a highly restricted and synchronized topography: MYO6-positive (MYO6(+)) cells were almost exclusively partitioned into the T cell and NK cell compartments, while remaining largely quiescent in other lineages (**Figures 8F–H**). This highly specific cellular distribution provided a robust rationalization to confine our downstream high-resolution analyses strictly within the isolated T/NK cell macro-environment, aiming to decrypt its compartment-specific biological relevance.

**Figure 9:**
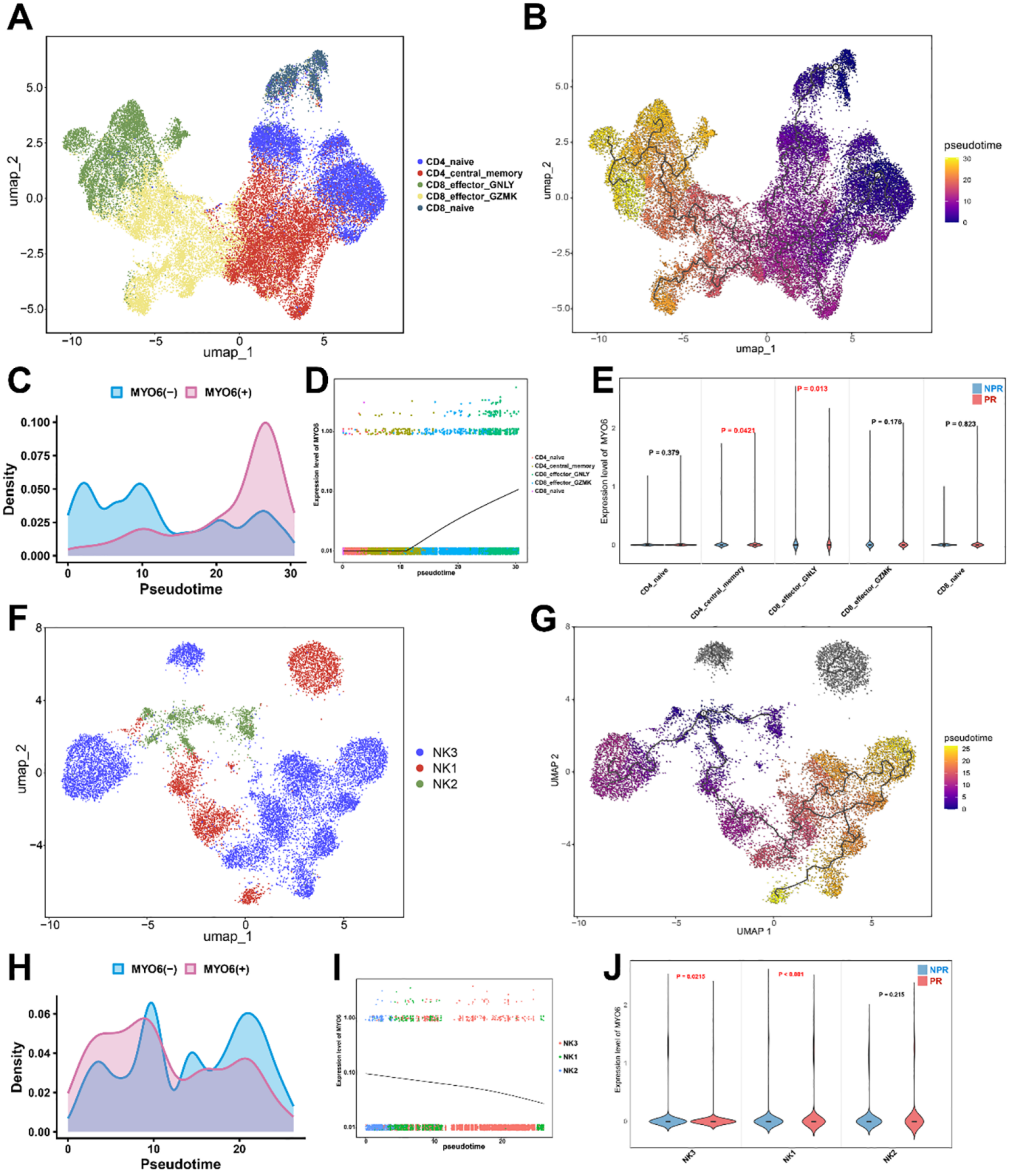
Single-cell subpopulation annotation and pseudo-time analysis of T/NK cells. **(A)** UMAP plot of annotated T cell subpopulations; **(B)** UMAP plot of T cell pseudo-time trajectories; **(C)** Density plot of MYO6(-)/MYO6(+) in T cell pseudo-time; **(D)** Line chart of T cell subpopulation gene expression dynamics along pseudo-time; **(E)** Box plot of MYO6 expression in T cell subpopulations (NPR vs PR); **(F)** UMAP plot of annotated NK cell subpopulations; **(G)** UMAP plot of NK cell pseudo-time trajectories; **(H)** Density plot of MYO6(-)/MYO6(+) in NK cell pseudo-time; **(I)** Line chart of NK cell subpopulation gene expression dynamics along pseudo-time; **(J)** Box plot of MYO6 expression in NK cell subpopulations (NPR vs PR);.

T cells and NK cells populations serve as the core immune cell subpopulations regulating inflammatory responses, myocardial injury repair, and disease progression within the MI immune microenvironment. To elucidate the expression specificity of MYO6 across T cell and NK cell subtypes and its regulatory role in the differentiation trajectories and functional state transitions of both cell types, we isolated T cells and NK cells separately for subpopulation annotation and pseudotime analysis. T cells were annotated into five subpopulations: CD4_naive, CD4_central_memory, CD8_effector_GNLY, CD8_effector_GZMK, and CD8_naive (**Figure 9A**). Pseudotime analysis revealed that the T-cell differentiation trajectories, originating from CD4_naive and CD8_naive cells, exhibit a tendency to differentiate from the initial state towards the memory/effector state (**Figure 9B**). MYO6 (+) and MYO6 (-) cells displayed heterogeneous distributions along the pseudotime axis, with MYO6 (-) cells predominantly clustered among early- pseudotime naive T cells, whilst MYO6 (+) cells exhibited peak density among late-pseudotime effector T cells. This indicates that T cells differentiating towards effector functional states under myocardial infarction conditions exhibit a pronounced tendency to express MYO6 (**Figures 9C, D**). Further comparison of MYO6 expression levels across T cell subsets between the NPR and PR groups revealed no significant alterations in their initial states. However, the NPR group exhibited markedly higher MYO6 expression in plaque-stabilizing CD4_central_memory cells compared with the PR group, whereas the PR group demonstrated elevated MYO6 expression in pro-inflammatory CD8_effector_GNLY cells (**Figure 9E**). These findings suggest that within the pathological microenvironment of MI, MYO6 is closely associated with the functional activation of T cells and may exert protective regulatory effects on plaque maintenance.

Drawing upon the NK cell classification system established by Rebuffet et al., which features unique surface markers and transcriptomic characteristics (15), we successfully distinguished NK1, NK2, and NK3 subsets. Specifically, the NK1 subset represents a cytotoxic effector state characterized by CX3CR1, FCGR3A, and CD38; the NK2 subset serves as a transitional, tissue-infiltrating population expressing CD27, NCAM1, ICAM1, CD44, and CD96; while the NK3 subset exhibits an immunoregulatory/immunosuppressive phenotype marked by KIR2DL3, IL2RB, ITGB2, ITGB7, and NGFR. The NK2 subset was identified as the initial state of NK cell differentiation, differentiating towards NK1 and NK3 lineages (**Figures 9F, G**). The distribution of MYO6 (+) and MYO6 (-) cells along the pseudo-time axis broadly exhibited an initial increase followed by a decrease. MYO6 (+) cells were predominantly enriched in the NK2 subset, while MYO6 (-) cells were primarily located in the differentiated NK1/NK3 regions (**Figures 9H, I**). Further comparison of MYO6 expression levels across NK cell subsets revealed that, compared with the NPR group, the PR group exhibited significantly elevated MYO6 expression in the NK1 subset and markedly reduced NK3 expression (**Figure 9J**). In summary, altered MYO6 expression may drive NK cell differentiation towards the highly cytotoxic NK1 subset while simultaneously attenuating the immunosuppressive function of the NK3 subset. This disrupts local plaque immune equilibrium, thereby promoting plaque rupture and exacerbating myocardial infarction.

**Figure 10:**
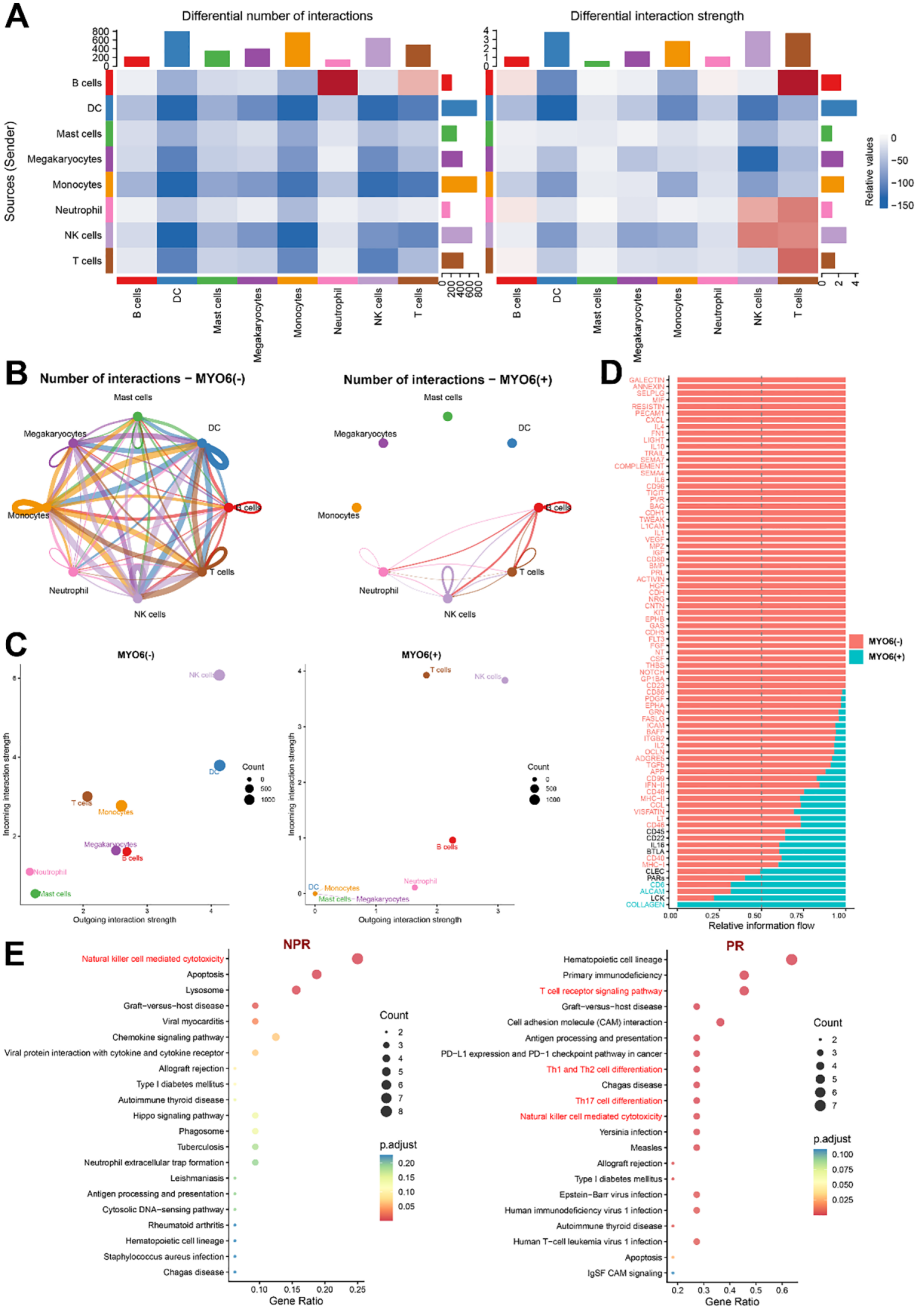
Single-cell cell communication analysis and KEGG analysis of MYO6 knockout differential genes. **(A)** Heatmaps of differential cell interaction number and strength; **(B)** Cell interaction networks of MYO6(-) and MYO6(+) groups; **(C)** Scatter plots of cell interaction strength in MYO6(-) and MYO6(+) groups; **(D)** Bar plot of relative information flow of ligand-receptor pathways; **(E)** KEGG pathway enrichment analysis of MYO6 knockout differential genes in NPR and PR groups.

To elucidate the regulatory role of MYO6 in intercellular communication among immune cells, we compared the intercellular communication characteristics between MYO6 (+) and MYO6 (-) cell populations. The results revealed that MYO6 expression suppresses intercellular communication activity between T cells and NK cells: in the MYO6 (-) state, both T cells and NK cells exhibited higher input/output communication intensity and greater communication frequency, indicating more frequent signal exchange between immune cells. Conversely, in the MYO6 (+) state, both the strength and quantity of communication in T cells and NK cells decreased. This reduction in excessive interaction and activation of immune cells suggests a protective role in the immune microenvironment by ‘regulating inflammation and maintaining homeostasis’ (**Figures 10A–C**). Further analysis of relative information flow characteristics in communication pathways between MYO6 (-) and MYO6 (+) cell populations revealed substantial differences in immune regulation and matrix remodeling-related pathways. Notably, the COLLAGEN pathway exhibited an exceptionally high proportion in the MYO6 (+) group (**Figure 10D**). Because the COLLAGEN pathway is central to extracellular matrix remodeling and post-injury tissue repair, enhanced intercellular communication via this pathway under MYO6 (+) conditions may accelerate tissue repair in the infarct region, further demonstrating the protective regulatory function of MYO6 in MI.

Finally, to elucidate differential responses in immune-related molecular pathways across distinct plaque states (NPR/PR) following MYO6 knockout, we performed KEGG pathway enrichment analysis on significantly altered genes post-knockout (**Figure 10E**). In the NPR group, pathways significantly enriched following MYO6 knockout were predominantly related to immune function. Notably, the significant enrichment of the natural killer cell-mediated cytotoxicity pathway suggests that, within the stable microenvironment of intact plaques, MYO6 knockout substantially impacts NK cell cytotoxicity-related signaling. In the PR group, pathways significantly enriched following MYO6 knockout included T cell receptor signaling, Th1 and Th2 cell differentiation, Th17 cell differentiation, and natural killer cell-mediated cytotoxicity – all core pathways for T/NK cell immune activation. Notably, the activation effect of MYO6 knockout on T cell signaling pathways was markedly stronger in the PR group than in the NPR group, suggesting that the modulation of T-cell signaling pathways by MYO6 serves as a key mechanism influencing plaque homeostasis in MI.

## 4 Discussion

MI, a cardiovascular emergency with high global incidence and mortality, exhibits a complex pathological progression and significant prognostic variability. Precise diagnosis and mechanism-targeted interventions are therefore pivotal for improving clinical outcomes (16). Accumulating evidence indicates that MI is not merely a myocardial ischaemic-necrotic disease; dynamic immune dysregulation permeates its entire course, from acute inflammatory infiltration and necrotic cell clearance to subacute immune homeostasis, as well as chronic-phase tissue repair and fibrotic remodeling (6, 17, 18). Although clinical practice commonly employs markers such as troponin and creatine kinase isoenzymes for MI diagnosis, they offer limited insight into the underlying pathological mechanisms, the state of the immune microenvironment or the risk of disease progression. This highlights the need for novel biomarkers that are mechanistically linked to MI pathogenesis. This study addresses this gap by employing a multidimensional analytical strategy to identify such biomarkers, providing new avenues for precise diagnosis, prognostic assessment, and immune-targeted therapies.

Through integrated bioinformatics and machine learning analyses, we initially identified 15 key genes that were consistently downregulated in MI patients and demonstrated robust diagnostic efficacy in both the training set. The majority of these genes are closely associated with immune regulation. First, core immune regulatory genes including FASLG, KLRF1, GZMB, PRF1, IL2RB, SH2D1B and PYHIN1 primarily govern immune cell activation, apoptosis, cytotoxicity, inflammatory cascades and antigen presentation (18–23). Second, immune-associated regulatory genes (such as PDGFD, CCL4 and SYTL2) exert their effects by modulating immune cell function, inflammatory signaling, and immune-related cellular processes (24–26). Additionally, Smad7, ENPP4, ENPP5, and AKR1C3 are primarily involved in fibrosis, nucleotide metabolism, and oxidative stress processes, which exhibit close cross-regulatory interactions with immune modulation (27–29). MYO6, an actin-based motor protein, participates in multiple critical cellular processes including endocytosis, polarized secretion, and autophagy (30). Multiple studies confirm that MYO6 protects cardiomyocytes by regulating myocardial autophagy and maintaining myocardial mitochondrial metabolism (31, 32). The foregoing findings demonstrate that an imbalance in immune homeostasis constitutes a core pathological driver of MI. These genes may synergistically regulate immune cell function, inflammatory signaling equilibrium, and immune homeostasis, thereby contributing to MI progression.

To further validate potential causal associations between these genes and the diseases while eliminating bias arising from confounding factors, we introduced MR analysis for an in-depth screening. The results revealed that among the 15 candidate genes, only MYO6 exhibited a significant negative correlation with myocardial infarction, suggesting that MYO6 may exert a protective regulatory role in the pathological progression of MI. Furthermore, given the central role of immune responses in MI, we identified MYO6 as a core immune-related research target in MI. Subsequent immune infiltration analysis revealed a significant enrichment of pro-inflammatory immune cells, including neutrophils, monocytes, and M0 macrophages in patients with MI, alongside a marked reduction in cell subsets that regulate immune homeostasis, such as resting natural killer cells and activated CD4 memory T cells. These findings align with previously reported immune imbalances characterized by the excessive infiltration of pro-inflammatory cells during the acute phase of MI (33). Correlation analysis between MYO6 expression and immune cell subsets revealed its regulatory direction: MYO6 exhibited significant positive correlations with immune cells including CD8 T cells, activated CD4 memory T cells, and resting NK cells, whilst showing negative correlations with pro-inflammatory cells such as neutrophils, monocytes, and M0 macrophages. This suggests that MYO6 may mitigate tissue damage during MI by alleviating immune dysregulation.

Single-cell RNA sequencing analysis provided deeper mechanistic insights. MYO6 exhibited cell-specific enrichment patterns with critical regulatory functions: within T cells, it was enriched in effector states and highly expressed in CD4_central_memory T cells, where it likely suppresses the excessive activation of pro-inflammatory CD8_effector_GNLY and CD8_effector_GZMK T cells; In NK cells, MYO6 was concentrated in the naive NK2 subset, helping to maintain a balanced phenotype by restraining differentiation toward highly cytotoxic NK1 cells and preserving the immunosuppressive function of NK3 subset. Furthermore, MYO6 prevents inflammatory storms triggered by immune cell ‘cross-activation’ by suppressing excessive intercellular communication in T and NK cells, while simultaneously enhancing the collagen pathway to promote tissue repair in the infarct area. Conversely, MYO6 deficiency markedly activates T and NK cell-associated immune activation pathways (particularly in the PR group, where T cell pathway activation is more pronounced), disrupting immune equilibrium and accelerating plaque rupture and MI progression.

Nevertheless, we must candidly acknowledge several inherent limitations of this study methodology. First, the publicly available scRNA-seq and bulk transcriptomic data utilized are retrospective in nature, which may introduce potential confounding factors and selection biases. Second, although four independent clinical cohorts provided robust cross-platform validation, the sample sizes within individual cohorts remained relatively limited; thus, future validation through rigorous, prospective, multicenter studies with expanded cohorts is highly warranted. Third, although the matrices employed yielded high-resolution insights into plaque vulnerability, the available public single-cell datasets were structurally limited by a lack of control samples from truly “healthy” individuals. To mitigate this, we strategically adopted NPR as an internal disease baseline, shifting our focus to the dynamic process of PR. Furthermore, we successfully conducted a supplementary bulk-level CIBERSORT immune infiltration analysis on a training set that explicitly included healthy individuals, which robustly confirmed the multi-omic alignment of T/NK cell dysregulation against a genuine healthy baseline and compensated for the single-cell constraint. Finally, although our animal experiments successfully demonstrated the pathogenic relevance of MYO6, we did not conduct *in vivo* single-cell multicolor flow cytometry or cell-sorting-based, cell-type-specific tracking to directly isolate the impact of MYO6 within the dynamic T/NK cell infarction microenvironment. Integrating standardized, large-scale, multicenter single-cell datasets alongside sorted-cell functional assays remains a top priority for our follow-up research to further refine these cellular resolution boundaries.

## 5 Conclusion

In summary, evidence from clinical samples confirms that MYO6 participates in the pathological regulation of myocardial infarction through multiple mechanisms. Specifically, it modulates immune system responses, guides the differentiation patterns of T and NK cell subsets, and simultaneously regulates intercellular communication and repair-related pathways. This synergistic regulatory mechanism underscores the pivotal role of MYO6 in maintaining immune homeostasis and stabilizing plaques during MI progression. Systematically elucidating of this mechanism not only clarifies the protective role of MYO6 in the pathogenesis of MI but also identifies it as a novel potential therapeutic target for clinical intervention focused on immune cell regulation. Collectively, these findings provide a robust theoretical foundation for the development of subsequent immune-targeted therapies for MI.

## Data Availability

The original contributions presented in the study are included in the article/supplementary material. Further inquiries can be directed to the corresponding authors.
