# Supplementary figures and images for: Uncovering the pivotal role of MYO6 in myocardial infarction: a multimodally validated diagnostic biomarker and immunotherapeutic target
Source: Front Immunol. 2026 Jul 9;17:1797028. doi: 10.3389/fimmu.2026.1797028 (PMC13391262; doi:10.3389/fimmu.2026.1797028)

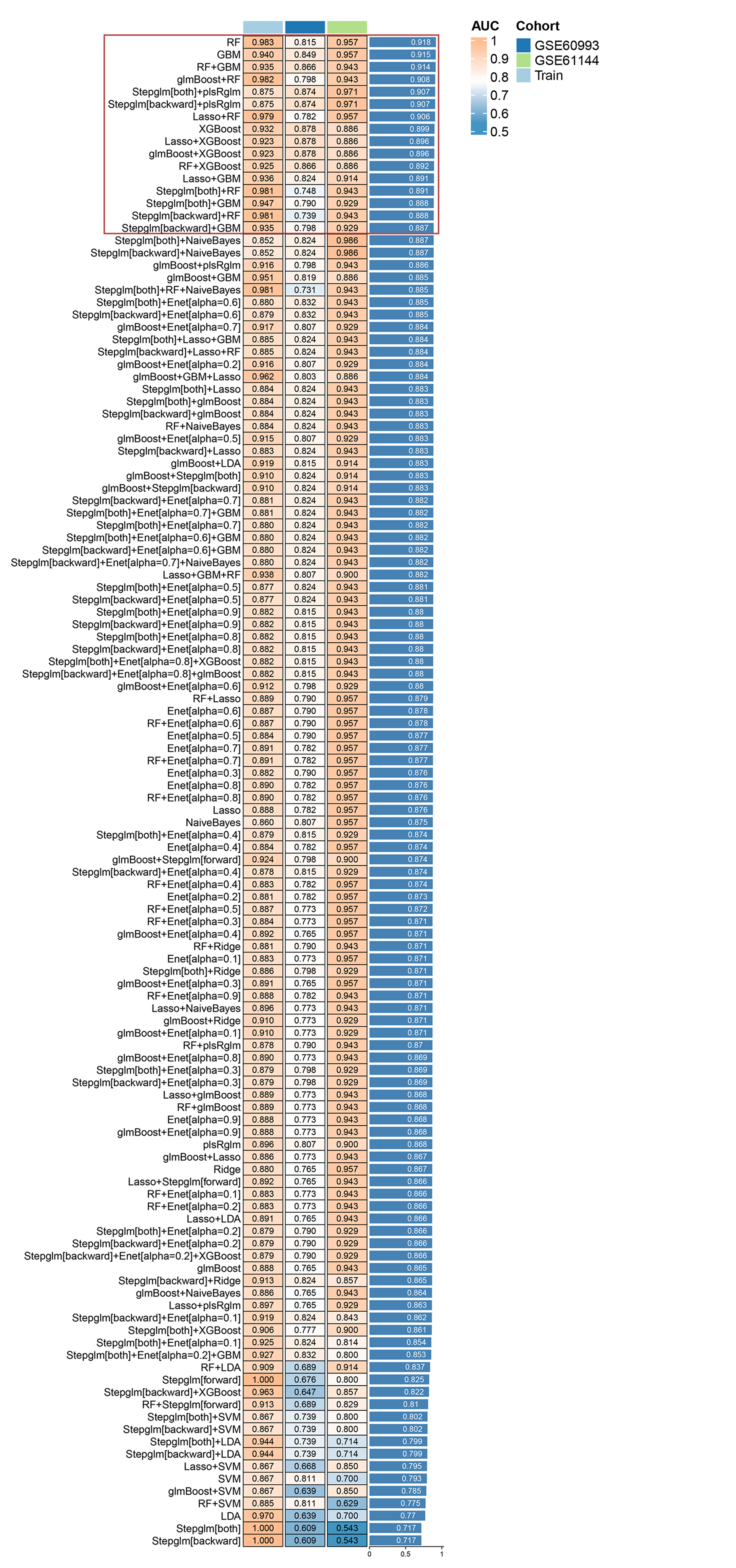

Supplement: Supplementary file 1 [file Image1.tif]
